# Assessing the dietary habits and nutritional status of secondary school students in Kraków and the Myślenice district (2016–2017): implications for public health

**DOI:** 10.3389/fpubh.2025.1592361

**Published:** 2025-08-07

**Authors:** Jaśmina Żwirska, Ewa Błaszczyk-Bębenek, Izabela Bolesławska, Paweł Jagielski, Agnieszka Ostachowska-Gąsior, Paweł Kawalec

**Affiliations:** ^1^Department of Nutrition and Drug Research, Institute of Public Health, Faculty of Health Sciences, Jagiellonian University Medical College, Kraków, Poland; ^2^Department of Bromatology, Poznan University of Medical Sciences, Poznan, Poland

**Keywords:** dietary habits, fast food products, nutrition, nutritional status, secondary school students, snacking

## Abstract

Adolescence is a period of rapid growth and development, making proper nutrition essential for overall health and fitness, as well as proper physical and cognitive development. The aim of this study was to assess consumption patterns of everyday food, fast food, and snacks among adolescents aged 12–16 years, depending on sex and nutritional status. The survey was conducted in the Myślenice district (a second-level local government unit) and in Kraków, Poland, between 2016 and 2017. It included secondary school students aged 12–16. The survey of children after obtaining consent from parents was conducted based on the validated FFQ questionnaire. Anthropometric measurements included body weight, height, body fat percentage, and waist and hip circumference. Depending on the BMI value, the children were divided into three groups–underweight, overweight and obese, and with normal body weight. Significant differences in the frequency of fast food consumption and snacking were observed between boys and girls, depending on BMI. The more frequent consumption of whole meal bread by overweight and obese adolescents, along with their lower intake of foods high in simple sugars and fast food, may suggest conscious attempts to control their diet, reflecting changing eating habits among young people. In contrast, the higher fast food consumption among underweight girls, compared to other groups, may suggest a potential link between low body weight and a tendency to consume foods with low nutritional value. Special attention should be given to snacking habits, as they play a significant role in shaping young people’s diet.

## 1 Introduction

Adolescence is a period of rapid growth and development, making proper nutrition essential for overall health and fitness, as well as proper physical and cognitive development. Adequate intake of essential nutrients, including vitamins and micronutrients, plays a crucial role in a young person’s performance at school. In addition to the energy value of meals, the timing and frequency of food consumption also significantly affect proper and effective nutrition (1).

Maintaining a healthy body weight depends on a balanced relationship between energy intake and expenditure (2–5). Both underweight and excess weight (overweight and unhealthy weight) increase the risk of numerous health conditions, negatively impacting both quality of life and life expectancy. In European countries, the prevalence of overweight and obesity among children is estimated at 10–20%, while in Poland, it reaches 18%. Addressing the growing problem of overweight and obesity from an early age is essential for long-term public health benefits (6, 7). Excess body weight also increases the risk of hypertension, hypercholesterolemia, stroke, sleep apnea, gallbladder disease and bronchial asthma. In children and adolescents, it is further linked to numerous psychological consequences such as low self-esteem, depression and suicidal thoughts. Genetic predisposition accounts for approximately 25–45% of obesity cases, while other risk factors include behavioral, environmental, socio-cultural and socioeconomic influences, primarily poor eating habits and low levels of physical activity. The challenges associated with obesity treatment, including high costs, highlight the need for effective preventive measures across the entire pediatric population, with particular attention to children from economically disadvantaged backgrounds and low socioeconomic status. Preventive measures should be implemented on global, national, regional and local levels to effectively combat overweight and obesity (8).

Malnutrition contributes to the early onset of diet-related chronic diseases, including cardiovascular disease, type 2 diabetes, cancer, obesity, and osteoporosis (9). In addition, unhealthy behaviors and poor dietary choices in childhood are strong predictors of adult health and are linked to an increased risk of chronic conditions such as obesity, cardiovascular disease, type 2 diabetes, and certain types of cancer (10). Moreover, eating habits formed in childhood and adolescence play a key role in shaping future food preferences, diet quality and body weight, making them essential for establishing lifelong health-promoting habits (11).

Nutritional behavior is shaped by a combination of physiological, environmental, psychological, cultural, socioeconomic and genetic factors (12). However, the complex interplay between these influences is not fully understood (10, 13). In highly developed countries such as Poland, non-economic factors play a particularly significant role in establishing dietary habits. These include environmental, psychological and socio-cultural factors such as education, occupation, lifestyle, customs, age, sex and place of residence (12). Research also suggests that nutritional knowledge impacts food preferences (14). While not all factors influencing eating habits can be modified, many can be shaped. Environmental exposure and food experiences play a crucial role in the development of eating behaviors in children and adolescents (15).

A key factor influencing human eating behavior is social modeling, where individuals look to others as a guide to what and how much to eat (16). When children enter school, they experience lifestyle and dietary changes, becoming highly receptive to new behaviors through imitation. During this period, the school environment begins to play a role as significant as that of parents (12, 16). Found that social modeling is especially strong when individuals seek to identify with a role model or perceive themselves as similar to them. This process is, at least in part, driven by unconscious behavioral imitation.

Children’s eating behavior is significantly related to parental education levels and family financial status. Higher education and income levels are associated with a lower incidence of eating disorders (17). Research indicates that fruit and vegetable consumption during youth has a protective effect against cancer. Conversely, the widespread availability of high-energy foods rich in sugar, fat and salt promotes dietary preferences that are not in line with nutritional guidelines, contributing to excessive weight gain, obesity and other diet-related diseases (17). In the context of children’s diets, the phenomenon of “McDonaldization” is reflected in the widespread availability and consumption of ultra-processed foods high in sugar, salt, and fat and low in nutritional value. These eating habits are emblematic of the Western dietary pattern, which has been associated with numerous adverse health outcomes in children and adolescents (18). Studies have shown that adherence to a Western diet increases the risk of obesity, insulin resistance, and other cardiometabolic disorders. A systematic review found that children following Western dietary patterns had higher body mass index (BMI), waist circumference, and glucose and triglyceride levels compared to those with healthier diets. In addition, high consumption of ultra-processed foods has been linked to poorer academic performance, with adolescents scoring lower in mathematics, reading, and writing (19). Early-life exposure to Western diets may also induce long-term changes in the gut microbiome, potentially affecting lifelong health. Moreover, a diet rich in fat and low in fiber—another hallmark of the Western pattern—was linked to increased rates of asthma and other allergic conditions in children (20).

The aim of this study was to assess consumption patterns of everyday food, fast food, and snacks among adolescents aged 12–16 years, depending on sex and nutritional status. The results of the study may constitute a valuable contribution to the development of obesity prevention strategies and nutritional education. Proper nutrition of a developing organism has a fundamental impact on its biological efficiency, learning capabilities and well-being. With this in mind, the results of the study may contribute to updating information on the nutritional status and eating habits of young people. Thanks to this, it will be possible to undertake appropriate educational activities for young people and their parents so that their eating behaviors are correct in the future. Obesity is not only a health problem, but also a social and economic one.

## 2 Materials and methods

### 2.1 Study description

The survey was conducted in the Myślenice district (a second-level local government unit) and in Kraków, Poland, between 2016 and 2017. It included secondary school students aged 12–16 and their parents. The list of schools in the Myślenice district was prepared and provided to us by the Myślenice District Office, while in Krakow, the survey included those schools that agreed to conduct the survey. Children were included in the study whose parents agreed to have their child surveyed and completed a questionnaire that included questions about parental education. Inclusion criteria for the study were as follows: (1) students aged between 12 and 16 years; (2) enrollment in a participating secondary school in the Myślenice district or Kraków; (3) provision of informed consent by a parent or legal guardian; and (4) availability of completed questionnaires from both the student and the parent, including information regarding parental education. Exclusion criteria included: (1) lack of parental consent for the child’s participation; (2) incomplete survey data from either the student or the parent; (3) participant withdrawal at any stage of the study; and (4) schools that declined to participate in Kraków. The 715 parents were offered to complete the questionnaire, of which 15 did not agree to the survey. The surveys among parents were conducted with the participation of the school principals, who helped to pass them on to parents during class meetings or through students. The questionnaire was filled out by students, parents only filled out questions about the birth certificate. Both parents and students filled out the questionnaire at home. In the end, 700 children started the survey (they answered the first question). Complete survey questionnaires (parents and children) were obtained from 664 participants. All necessary consents were obtained from school principals and parents through the Department of Promotion and Health Protection at the District Office in Myślenice.

The study was approved by the Bioethics Committee of Jagiellonian University (no. K/ZDS/007167) and was conducted with the consent of school authorities, legal guardians, and the children themselves. It included both a questionnaire survey and anthropometric measurements. Participation in the study was voluntary, and participants could withdraw from the study at any stage. Participants were informed that anonymity and the aims and scope of the study would be preserved, and they gave informed consent to participate.

### 2.2 Questionnaire surveys

The survey questionnaire used in the study consisted of 29 questions, consisted of a section based on the Eating Habits and Eating Beliefs Questionnaire developed by the Behavioral Nutrition Team of the Committee on Human Nutrition of the Polish Academy of Sciences, approved and recommended for research in the Polish population (21), as well as a set of original, author-designed survey questions. No formal validation of the survey instrument and reliability testing was done. Both in the Myślenice district and Kraków, children without parental consent were excluded from participation. However, no child was excluded due to numerical limitations, ensuring that no participant felt ‘inferior’ or excluded for unknown reasons. Parents were informed about the purpose and methodology of the study by teachers and staff from the District Office. The parental survey included socio-economic questions concerning the family.

Parents were informed about the purpose and method of conducting the tests by teachers and employees of the District Office. The survey for parents included questions: socio-economic questions regarding the family. The survey questionnaire completed by the children included questions about the number and regularity of meals during the day, snacking between meals and the frequency of food consumption for selected products and beverages with possible answers and scoring: (1 = does not eat, 2 = less often, 3 = once a month, 4 = 1–2 times a week, 5 = 3–4 times a week, 6 = every day).

The questionnaires were distributed with the assistance of school authorities, who facilitated their delivery to parents during class meetings or through students. Teachers informed parents about the purpose and methodology of the survey.

### 2.3 Anthropometric measurements

Anthropometric measurements included body weight, height, body fat percentage, and waist and hip circumference. These measurements were conducted only for children who did not object and whose parents had provided consent for their participation. Measurements were taken in the school nurse’s office or a designated room, with boys and girls assessed separately. The procedure followed the guidelines of the Institute of Mother and Child (22, 23).

Body weight and body fat percentage were measured using a TANITA BF 556 electronic scale with an accuracy of 0.1 kg. Waist and hip circumference was measured with a measuring tape accurate to 1 mm, while height was recorded with a TANITA height meter.

Body mass index (BMI) was calculated for each child based on their measurements. Height, weight and BMI were then compared to centile charts from the OLAF project (24). Children with a BMI above the 95th percentile for their age and sex were classified as obese, while those with a value between 85th and 95th percentile were considered overweight. A BMI between the 15th and 85th percentile was categorized as normal weight. Underweight was defined as a BMI between the 5th and 15th percentile, while a BMI below the 5th percentile indicated significant underweight.

### 2.4 Statistical analysis

For statistical analysis, all data were encoded in Excel 2010 and imported into IBM SPSS Statistics version 25. The study population was described using numbers and percentages of people with a given characteristic. Quantitative characteristics were presented as mean and standard deviation. To assess differences between the groups of children based on sex and BMI, the non-parametric Kruskal-Wallis analysis of variance (for many independent samples) was used. The chi^2^ test was used to analyses qualitative variables. Additionally, BMI was calculated and compared to centile grids from the OLAF project. The level of statistical significance was set at *α* = 0.05.

## 3 Results

Of the 664 children surveyed, 314 were boys (47.3%) and 350 were girls (52.7%). The mean age of the participants was 13.85 ± 0.78 years. Among mothers, the most common level of education was secondary (40.5%), followed by higher education, vocational education and primary education. Among fathers, the highest proportion had vocational education (39.2%), followed by secondary education, university education and primary education (Table 1).

**Table 1:** Characteristics of children’s demographic features by gender.

Population characteristics	General (*n*) %	Boys (*n*) %	Girls (*n*) %
**Age (Years) X ± SD**	***N* = 664**	***N* = 314**	***N* = 350**
	13,8 ± 0,78	13,9 ± 0,78	13,8 ± 0,78
**Mother’s education**	***N* = 640**	***N* = 300**	***N* = 340**
Primary education	(14) 2.2	(8) 2.7	(6) 1.8
Elementary vocational	(138) 21.6	(57) 19.0	(81) 23.8
Secondary education	(259) 40,5	(128) 42.7	(131) 38.5
Higher vocational	(45) 7.0	(23) 7.7	(22) 6.5
Higher	(184) 28.8	(84) 28.0	(100) 29.4
**Father’s education**	***N* = 623**	***N* = 289**	***N* = 334**
Primary education	(25) 4.0	(13) 4.5	(12) 3.6
Elementary vocational	(244) 39.2	(112) 38.8	(132) 39.5
Secondary education	(212) 34.0	(94) 32.5	(118) 35.3
Higher vocational	(57) 9.1	(29) 10.0	(28) 8.4
Higher	(85) 13.6	(41) 14.2	(44) 13.2
**Mother’s education type**	***N* = 508**	***N* = 247**	***N* = 261**
Technical	(190) 37.4	(91) 36.8	(99) 37.9
Humanities	(114) 22.4	(54) 21.9	(60) 23.0
Biological medicine	(64) 12.6	(31) 12.6	(33) 12.6
Economics	(140) 27.6	(71) 28.7	(69) 26.4
**Father’s education type**	***N* = 497**	***N* = 239**	***N* = 258**
Technical	(417) 83.9	(204) 85.4	(213) 82.6
Humanities	(30) 6.0	(13) 5.4	(17) 6.6
Biological medicine	(7) 1.4	(2) 0.8	(5) 1.9
Economics	(43) 8.7	(20) 8.4	(23) 8.91

N, number of subjects; n, number of subjects in subgroup; X ± SD, mean ± standard deviation.Bold values indicate the total number of responses for each category (e.g., number of people, % of participants).

Significant differences were observed between boys and girls in the frequency of consumption of selected food products, fast food, and snacks, depending on BMI. The mean frequency of consumption for these products, categorized by sex and BMI, is presented in Table. Overweight and obese boys and girls consumed wholemeal bread significantly more often than their normal-weight and underweight peers (Table 2). Significant differences were also noted in the consumption of wheat bread, butter, sugar, crisps, potato crisps and savory snacks, with overweight and obese girls consuming these products significantly less often than normal-weight and underweight girls (*p* < 0.05). Additionally, both overweight and obese boys and girls ate sweets significantly less frequently than adolescents with normal weight or underweight (Table 2).

**Table 2:** Frequency of selected food product consumption in the study group by sex and BMI.

Product	Underweight (A)		Normal weight (B)		Overweight and obesity (C)		ANOVA Kruskal-Wallis rank *p*
	C X ± SD	D X ± SD	C X ± SD	D X ± SD	C X ± SD	D X ± SD	
Goats, pasta, rice	4.1 **±** 1.1	4.4 **±** 0.8	4.2 **±** 0.8	4.3 **±** 0.9	4.0 **±** 0.7	4.1 **±** 0.9	ABC-C 0.1702 ABC-D 0.1249
Bread (bread, wheat rolls)	5.6 **±** 1.0	5.9 **±** 0.5	5.8 **±** 0.6	5.6 **±** 0.9	5.5 **±** 1.1	5.2 **±** 1.4	ABC-C 0.0505 ABC-D **0.0059**
Wholemeal bread	2.9 **±** 1.8	3.7 **±** 1.6	3.3 **±** 1.7	3.5 **±** 1.6	4.0 **±** 1.6	4.1 **±** 1.6	ABC-C **0.0022** ABC-D **0.0330**
Milk	5.1 **±** 1.2	4.9 **±** 1.0	5.0 **±** 1.2	4.8 **±** 1.2	4.5 **±** 1.4	4.3 **±** 1.5	ABC-C **0.0116** ABC-D 0.1862
White cheese	3.4 **±** 1.2	3.4 **±** 1.1	3.3 **±** 1.3	3.5 **±** 1.3	3.5 **±** 1.4	3.8 **±** 1.2	ABC-C 0.3778 ABC-D 0.1475
Yellow cheese	4.7 **±** 0.8	4.3 **±** 1.2	4.5 **±** 1.2	4.2 **±** 1.2	4.3 **±** 1.3	4.2 **±** 1.3	ABC-C 0.3791 ABC-D 0.8913
Various yoghurts	4.2 **±** 1.1	4.5 **±** 1.0	4.3 **±** 1.3	4.4 **±** 1.1	4.0 **±** 1.2	4.2 **±** 1.5	ABC-C 0.3500 ABC-D 0.5947
Fish and fish products	3.4 **±** 0.9	3.4 **±** 1.0	3.3 **±** 1.0	3.3 **±** 0.9	3.4 **±** 1.0	3.4 **±** 1.0	ABC-C 0.8718 ABC-D 0.7467
Poultry	0.9 **±** 0.8	4.3 **±** 1.0	4.4 **±** 0.7	4.3 **±** 0.8	4.4 **±** 0.6	4.2 **±** 1.0	ABC-C 0.7289 ABC-D 0.8272
Red meat	3.6 **±** 1.2	3.2 **±** 1.2	3.6 **±** 1.2	3.2 **±** 1.2	3.4 **±** 1.2	2.9 **±** 1.2	ABC-C 0.3018 ABC-D 0.3939
Eggs	4.2 **±** 0.7	4.0 **±** 0.9	3.9 **±** 1.0	4.0 **±** 1.0	3.9 **±** 1.0	4.1 **±** 0.9	ABC-C 0.4059 ABC-D 0.8267
Vegetable oils, margarine	4.7 **±** 1.2	4.2 **±** 1.6	4.2 **±** 1.6	4.2 **±** 1.6	4.0 **±** 1.6	4.2 **±** 1.4	ABC-C 0.0617 ABC-D 0.9954
Butter	5.1 **±** 1.4	5.3 **±** 1.2	4.9 **±** 1.5	4.6 **±** 1.7	5.1 **±** 1.6	4.1 **±** 1.9	ABC-C 0.0884 ABC-D **0.0009**
Raw vegetables (vegetable juices)	4.3 **±** 1.5	4.0 **±** 1.4	4.0 **±** 1.5	4.1 **±** 1.5	4.0 **±** 1.6	4.4 **±** 1.4	ABC-C 0.3372 ABC-D 0.3646
Cooked vegetables	3.7 **±** 1.5	3.9 **±** 1.3	3.7 **±** 1.4	4.0 **±** 1.4	3.9 **±** 1.3	4.0 **±** 1.3	ABC-C 0.7483 ABC-D 0.9325
Raw fruit and fruit juices	5.1 **±** 1.3	5.3 **±** 0.8	5.0 **±** 1.1	5.3 **±** 1.0	5.0 **±** 1.1	5.5 **±** 0.7	ABC-C 0.6987 ABC-D 0.4662
Legume seeds	3.3 **±** 1.1	3.2 **±** 1.0	2.8 **±** 1.0	2.9 **±** 1.0	2.9 **±** 1.2	3.1 **±** 0.9	ABC-C **0.0099** ABC-D 0.1314
Unsalted nuts	2.4 **±** 1.0	3.0 **±** 1.0	2.5 **±** 1.1	2.6 **±** 1.1	2.3 **±** 1.1	2.6 **±** 1.0	ABC-C 0.5861 ABC-D 0.0865
Sweets	5.0 **±** 1.0	5.1 **±** 1.0	4.9 **±** 1.0	4.7 **±** 1.1	4.5 **±** 1.1	4.5 **±** 1.1	ABC-C **0.0221** ABC-D **0.0156**
Sugar	5.3 **±** 1.5	5.6 **±** 0.8	5.2 **±** 1.3	5.1 **±** 1.3	5.0 **±** 1.4	4.7 **±** 1.6	ABC-C 0.2568 ABC-D **0.0020**
Chips, crisps, savory sticks	3.7 **±** 1.0	3.9 **±** 1.2	3.8 **±** 1.2	3.6 **±** 1.1	3.6 **±** 1.0	3.2 **±** 1.0	ABC-C 0.4245 ABC-D **0.0183**

X ± SD, mean ± standard deviation; p, significance level; C – boys, D – girls, A – underweight, B – normal weight, C – overweight and obesity; The frequency of consumption scale: 1- does not eat, 2- less often, 3- once a month, 4-1-2 times a week, 5–3-4 times a week, 6- every day.Bold values indicate statistically significant results (*p* < 0.05).

No significant differences in BMI-related consumption patterns were observed between boys and girls for cereals, pasta, rice, white cheese, yellow cheese, yoghurt, fish, poultry, red meat, eggs, vegetable oils, margarines, vegetables, fruit, fruit juices and unsalted nuts. Fruit was consumed by 85.2% of overweight and obese girls and 65.7% of underweight and normal-weight boys. Overweight and obese boys ate fruit significantly less often than their normal-weight and underweight counterparts. They were also significantly less likely to consume pastries than boys with a normal weight or underweight (Table 3). In the underweight category, sweets were consumed by 73.5% of girls and 65.7% of boys, while in the overweight and obesity category, they were consumed by 42.6% of girls and 46.0% of boys. Overweight and obese girls consumed significantly less often sweets and significantly more often fruit compared to normal-weight and underweight girls (Table 3).

**Table 3:** Consumption of selected food products in the study group by sex and BMI.

Product	Answers	Underweight (A)		Normal weight (B)		Overweight and obesity (C)		Chi-square p	
		C (%)	D (%)	C (%)	D (%)	C (%)	D (%)		
Sweets	Yes	65.7	73.5	58.3	54.7	46.0	42.6	ABC-C	0.1157
	No	34.3	26.5	41.7	45.3	54.0	57.4	ABC-D	**0.0064**
Bread confectionery	Yes	31.4	20.4	19.0	15.4	7.9	11.1	ABC-C	**0.0131**
	No	68.6	79.6	81.0	84.6	92.1	88.9	ABC-D	0.4268
Fruit	Yes	65.7	61.2	65.7	76.5	46.0	85.2	ABC-C	**0.0159**
	No	34.3	38.8	34.3	23.5	54.0	14.8	ABC-D	**0.0156**
Raisins	Yes	2.9	0	1.9	4.0	3.2	3.7	ABC-C	0.7934
	No	97.1	100.0	98.1	96.0	96.8	96.3	ABC-D	0.3608
Crisps. Nuts	Yes	34.3	24.5	31.5	17.0	23.8	9.3	ABC-C	0.4352
	No	65.7	75.5	68.5	83.0	76.2	90.7	ABC-D	0.1186
Yoghurt. Cream cheese	Yes	57.1	59.2	49.1	49.4	44.4	42.6	ABC-C	0.4838
	No	42.9	40.8	50.9	50.6	55.6	57.4	ABC-D	0.2389
Corn chips	Yes	17.1	10.2	15.3	13.0	11.1	13.0	ABC-C	0.6463
	No	82.9	89.8	84.7	87.0	88.9	87.0	ABC-D	0.8648

C – boys, D – girls, A – underweight, B – normal weight, C – overweight and obesity, p – significance level.Bold values indicate statistically significant results (*p* < 0.05).

**Figure 1:**
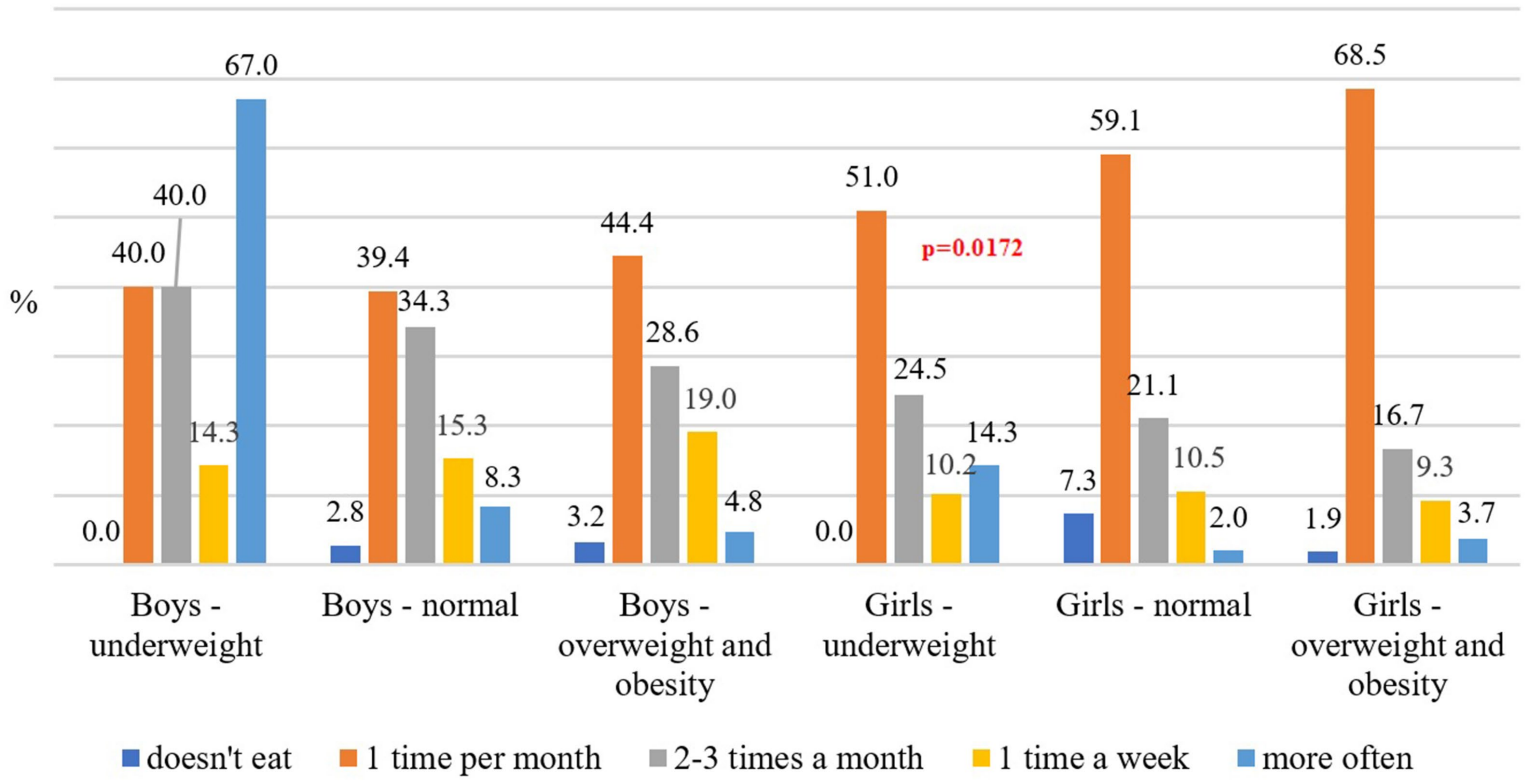
Frequency of fast-food consumption depending on sex and BMI.

Fast food was consumed once a month by 68.5% of overweight and obese girls and 44.4% of overweight and obese boys. No significant differences in fast food consumption were observed among boys across BMI categories. In contrast, underweight girls consumed these products significantly more often than both overweight and obese boys and girls (Figure 1).

## 4 Discussion

Eating habits are established in childhood, with a school-age child’s dietary choices largely shaped by their environment, particularly parental behavior. Children observe family members’ eating habits, including whether they follow hygiene practices, share meals at the table or engage in other activities such as reading, watching TV or listening to the radio while eating. While parents determine what their child eats, the child ultimately decides how much to consume. The family plays a crucial role in shaping food preferences, and the home has been identified as the primary source of nutritional knowledge (25).

A healthy diet enhances physical performance, increases stress resilience and supports immune function. It also helps maintain optimal total cholesterol and triglyceride levels, regulates carbohydrate metabolism and promotes a healthy body weight (26, 27). Poor nutrition and lack of physical activity play a key role in the development of various diseases, including obesity, diabetes, cardiovascular diseases, postural defects and osteoporosis (28, 29). In recent years, the prevalence of overweight and obese children and adolescents has risen globally, affecting both developed and developing countries (14, 30, 31).

Preliminary findings from the international HBSC study on the health behaviors of young people aged 11–15, in which Poland has participated for many years, indicated that excess body weight affects 29.7% of boys and 14.3% of girls (based on World Health Organization criteria, 2007). These percentages are slightly higher than those reported in the 2014 edition of the study. Between 2014 and 2018, the prevalence of overweight among young people increased from 19.9 to 21.7%, with a more pronounced increase among boys compared to girls, particularly in 13-year-olds of both sexes (32, 33).

The development of overweight and obesity among children and adolescents is strongly influenced by technological advancements and improved economic conditions. An increasing number of young people lead sedentary lifestyles, spending leisure time passively in front of computers or TV screens, which reduces physical activity. Additionally, a high-energy diet, along with excessive consumption of fast food, sweets and highly sweetened drinks, contributes to a positive energy balance and weight gain (9, 11, 23, 34–36).

A varied diet is essential to meeting the body’s nutritional needs. A Polish study on the health behaviors of secondary school students in the Pomeranian Voivodeship found that white meat was consumed an average of 2.4 times a week, while red meat was eaten twice a week. Legumes and fish appeared in the respondents’ diets only once per week (37). Maintaining a healthy body weight during adolescence is crucial, as research estimates that 70–80% of adolescents diagnosed with obesity will remain obese in adulthood (38). This is supported by findings from a study by Krawczyńska et al., who reported that among adolescents aged 16–18 attending vocational school, overweight and obesity were the most prevalent health concerns, affecting 11.68% of students (39).

A national nutrition survey found that bread is a fundamental component of the diet of young people aged 10–17. White wheat bread was the most commonly consumed, with 18% of boys and 21.7% of girls eating it once a day, and 27.3% of boys and 23.6% of girls consuming it several times a day. Whole meal or seeded brown bread and white rye bread were consumed less frequently, with most respondents reporting intake between 1–3 times a month and 1–3 times a week. A significant proportion did not consume these products at all: 30.4% of boys and 26.7% of girls did not eat dark bread, while 24.5% of boys and 24.1% of girls did not eat light rye bread. Breakfast cereals were relatively popular, with 10.1% of boys and 9.3% of girls consuming them at least once a day. Most respondents ate cereals 2–3 times a week (30.2% of boys and 26.3% of girls) or once a week (22.3 and 21.2%, respectively). Pasta and rice were consumed by nearly all participants. Fruit is another essential component of the daily diet; however, the survey revealed that fruit consumption was lower than recommended across all age groups of children and adolescents (40).

Bielaszka et al. (41) reported that overweight and obese boys and girls were significantly more likely to eat whole meal bread than their normal-weight and underweight peers. Dark bread, which is a source of dietary fiber and numerous minerals and vitamins, is not consumed frequently enough by schoolchildren. The authors found that only 8% of children ate dark bread daily, while 49% consumed it less than several times a week. Significant differences were also observed in the consumption of wheat bread, butter, sugar, crisps, chips and salty sticks. Overweight and obese girls consumed these products significantly less often than girls with normal weight and those who were not overweight. Similarly, both overweight and obese boys and girls ate sweets significantly less often than their normal-weight and non-overweight peers. Crisps, corn-based extruded snacks and other savory snacks remain highly popular, especially among younger generations. However, these products can be a potential source of acrylamide, a toxic compound that may form during frying and baking (42). Studies have shown that consuming crisps is associated with higher levels of anxiety, depression and emotional stress, as well as increased somatic symptoms, cognitive difficulties and fatigue compared to eating fruit (43). It is recommended to limit the sweetening of food for children and adolescents and to avoid sweets whenever possible. However, even the youngest children frequently consumed foods and drinks with added sugar, sometimes multiple times a day. The problem was even more pronounced in older age groups, where a clear increase was observed in the percentage of individuals adding sugar to food and drinks several times a day. In addition, children and adolescents were generally unlikely to avoid sweets, with most consuming them at least once a week or more often. The self-administered survey did not reveal any significant differences between boys and girls in BMI-related consumption patterns of groats, pasta, rice, white cheese, yellow cheese, yoghurt, margarine and fruit juices. Children and adolescents primarily consumed vegetable oils and butter in accordance with dietary recommendations. However, some respondents reported excessive butter consumption, with intake occurring several times a day (40). Our study also found no significant differences between boys and girls in BMI-related consumption patterns of vegetable oil.

Meat is an important source of nutrients in children’s diets, and it is recommended to consume lean meat, particularly poultry. In the Polish nutrition survey, poultry was the most commonly consumed meat among children and adolescents (40). However, pork, which has a higher fat content, was also relatively prevalent in their diet. Both younger and older children should primarily consume boiled or roasted meat, while limiting the intake of processed products. Despite this recommendation, a significant proportion of the respondents across all age groups consumed sausages relatively frequently (40). Our study found no significant differences between boys and girls in BMI-related consumption patterns of poultry and red meat.

Fish is an important source of high-quality protein, vitamins A (retinol), D and E, iodine, selenium and fat, especially long-chain n-3 polyunsaturated fatty acids. Increasing evidence suggests that fish consumption supports child development and learning, promotes eye health, and protects against cardiovascular disease and certain types of cancer (44). Eggs are another nutritionally valuable food, offering a range of health benefits. Their components have been associated with antimicrobial, anti-adhesive, immunomodulatory, antitumor, antihypertensive and antioxidant effects. Eggs also contain protease inhibitors, essential nutrients and functional lipids, highlighting their importance in human health, disease prevention and treatment. In our study, no significant differences were observed between boys and girls in BMI-related consumption patterns of fish, unsalted nuts and eggs. Although nuts are high in calories, they contain fatty acids, vegetable proteins, fiber, vitamins, minerals, carotenoids and phytosterols, which have potential antioxidant effects (45).

Fruit and vegetables are widely recognized as health-promoting foods due to their numerous compounds associated with a reduced risk of cardiovascular disease, obesity and certain types of cancer (46, 47). They are also rich in vitamins and minerals and serve as a source of phytochemicals, which exert protective effects through antioxidant, phytoestrogen and anti-inflammatory properties (48). The fiber found in vegetables, fruit and whole grains helps increase satiety after meals, reducing cravings for unhealthy foods and sugar. Additionally, fiber intake is negatively correlated with the risk of chronic diseases such as obesity, type 2 diabetes, cancer and cardiovascular disease. While the precise mechanisms underlying fiber’s role in metabolic health are not fully understood, it is believed to influence nutrient absorption, transit rate, and the production of short-chain fatty acids and gut hormones by altering intestinal viscosity (49). Our study found no significant differences between boys and girls in BMI-related consumption patterns of fruit and vegetables.

Kiciak et al. (50) found that nearly half of the respondents consumed fruit and vegetables several times a day. Approximately 25% of girls ate fruit and vegetables 1–2 times a day, compared to 20% of boys. The proportion of young people who consumed fruit and vegetables several times a week ranged between 23 and 28% (50).

Sochacka-Tatar and Stypuła (50) found that most teenagers consumed snacks between meals, with 38.8% reporting regular snacking. This behavior was most common among students attending technical secondary school and least common among middle school students. Nearly 95% of the respondents frequently ate crisps or sweets, with secondary school students consuming these products significantly more often. Fast food consumption was also widespread, as only 12.9% of the respondents reported not eating fast food at all (51). In our study, 68.5% of overweight and obese girls and 44.4% of overweight and obese boys consumed fast food once a month. While no significant differences were observed in fast food consumption among boys, malnourished girls consumed these products significantly more often. The frequent consumption of fast food among malnourished or food-insecure adolescent girls is influenced by a combination of economic hardship, accessibility, and psychosocial factors. The observed higher consumption of fast food among underweight girls is indeed intriguing; however, the discussion does not address potential structural determinants, such as food insecurity. International research has shown that underweight status can coexist with high intake of inexpensive, highly processed foods, particularly in economically disadvantaged populations. In such contexts, limited financial resources may lead individuals to rely on energy-dense but nutrient-poor foods, which, despite their caloric content, may not support adequate weight gain or nutritional adequacy. Including such socioeconomic considerations would provide a more nuanced understanding of the findings and could inform more targeted public health interventions (52, 53).

Wojtyła et al. found that 77% of adolescents snacked between meals, with 25% also snacking at night, most often on high-calorie foods. However, a significant proportion of secondary school students also consumed fruit between main meals (54). In our study, a higher percentage of overweight and obese girls compared to underweight and normal-weight boys snacked on fruit, while overweight and obese boys were significantly less likely to do so. Pastries were consumed more frequently by boys and girls with malnutrition, with overweight and obese boys consuming them significantly less often than their normal-weight and malnourished counterparts. Sweets were consumed by a large proportion of malnourished girls and boys, whereas overweight and obese girls and boys snacked on sweets less frequently. Additionally, overweight and obese girls were significantly less likely to snack on sweets and significantly more likely to snack on fruit compared to normal-weight and malnourished girls. This may indicate a conscious attempt by this group to control their diet.

In the study by Mikulec et al. (55) the epidemic situation, according to 50.0% of boys and 62.7% of girls, influenced their eating behavior. There were no significant differences in the number of meals consumed before the pandemic (*p* = 0.43) and during it (*p* = 0.12) between boys and girls. Before the pandemic, people eating 3 meals dominated in both groups (47.1% of boys and 45.2% of girls). During social isolation, the number of people eating 5 or more meals a day increased in both groups by 13.2 percentage points (boys) and by 10.5 percentage points (girls), and the number of people eating 3 meals decreased by 11.8 percentage points (boys) and by 15.2 percentage points (girls). A significant difference was observed in the regularity of eating meals during the pandemic between girls and boys. Among girls, 62.3% and among boys 48.0% declared that they did not eat meals regularly during the pandemic. There was no significant difference in the frequency of meals and increased appetite between girls and boys. Similar results were observed by Pietrobelli et al. (56) who observed an increase in the number of meals consumed more often among boys than girls.

Previous research has shown that most young people have unhealthy eating habits, including a monotonous diet lacking nutritionally rich foods. These habits contribute to abnormal development and an increased risk of eating disorders (57). Numerous studies have also demonstrated a link between children’s and adolescents’ eating habits and parental education levels. Children of parents with higher education were less likely to experience dietary problems. Low fruit and vegetable consumption has been associated with limited availability of these products and lower educational status (58–60). Kazimierczak et al. (61) and Wojtyniak et al. (62) found that individuals with higher education are more likely to consume fruit and vegetables. In our survey, mothers most commonly reported having secondary education, while primary education was the least frequently declared. Among fathers vocational education was the most common, whereas primary education was the least reported.

School education plays a key role in teaching young people about health and helping them apply these skills in practice. However, research indicates that students’ health behaviors are not adequately shaped. To eliminate unhealthy habits, it is essential to promote self-esteem and a sense of responsibility for one’s own health. This growing concern highlights the need for initiatives that promote a healthy lifestyle and educate children and adolescents on proper nutrition and physical activity. Educational activities targeting parents and adolescents play an important role in shaping healthy eating habits and often have positive short-term outcomes. In the long term, such efforts should contribute to expanding health knowledge, fostering pro-health attitudes, and developing practical skills along with appropriate hygiene and health-related habits. Children and adolescents acquire basic nutritional knowledge primarily at home by observing the behavior of household members and through direct guidance from parents. Family eating habits have a statistically significant influence on adolescents’ nutritional awareness. Further education occurs at school, where students’ eating behaviors can be shaped through classroom instruction, the types of meals provided, and the offerings of the school shop (63).

Attention is now being drawn, not only to the problem of the supply of energy (carbohydrates and fats) and protein in food, but additionally to two other dimensions, i.e., the deficit of micronutrients (micronutrients, vitamins), and the occurrence of overweight and obesity. In this context, the phenomenon of Double Burden of Malnutrition (DBM), consisting in the simultaneous occurrence of nutritional deficiencies and overweight or obesity, is increasingly identified. At the same time, the concept of triple burden or even multiple burden of malnutrition is emerging in the literature, which refers to the overlap of different forms of malnutrition - both quantitative and qualitative - within a population, household or even an individual. The rapid increase in the prevalence of overweight and obesity is directly linked to dynamic changes in food systems - in particular the increasing availability of cheap, ultra-processed foods and sweetened beverages, as well as a significant reduction in physical activity. Ultra-processed foods, an essential component of the modern diet, provide significant amounts of calories but lack dietary fiber, vitamins and minerals, which not only promotes the development of obesity but also exacerbates micronutrient deficiencies. In addition, reduced physical activity reduces energy expenditure. These deficiencies may also result from disturbances in the absorption, distribution and excretion of nutrients. In addition, chronic low-grade inflammation accompanying obesity can lead to abnormal micronutrient metabolism, which exacerbates deficiencies despite apparently sufficient energy intake (64). Although DBM mainly affects low- and middle-income countries (65), a similar problem is also observed in highly developed countries (66). Children and adolescents who are exposed to malnutrition (both in terms of energy and quality) develop less well, have learning difficulties and are more likely to fall ill. I In addition to long-term chronic health effects, malnutrition also has its day-to-day consequences, such as fatigue, apathy, lack of energy, disruption of processes related to brain function. Endocrine disorders are also common in such cases, which develop in secret for a long time, and one of the first visible symptoms can be menstrual disorders in girls (67).

There is currently a discussion in the world about food security after 2015. This time caesura is related to the fact that 2015 was adopted as the time point for achieving the goals of reducing hunger in the world. The challenge of the world society is to solve the problems of hunger and malnutrition in the world. However, it turns out that malnutrition is a deeper and broader problem than just consuming the right amount of energy and protein, which has been the main focus so far, and which was reflected in the concept of the so-called “undernourishment,” i.e., consuming dietary energy for an active and healthy life. Currently, many micronutrients important for human health have been identified, but most of them are not widely measured. Three of the commonly measured micronutrient deficiencies and related disorders refer to the lack of vitamin A, iron (anemia) and iodine (68).

Both excess and deficiency of nutrients in daily food adversely affect a child’s development. An imbalance between the energy value of consumed food and energy expenditure can lead to malnutrition, overweight and obesity. Malnutrition can be caused by the supply of foods with low energy and nutritional value, but at the same time by an increase in the body’s energy demand. Children and adolescents exposed to malnutrition (both in terms of energy and quality) develop worse, have problems with learning, and fall ill more often. The lack of appropriate vitamins and microelements in the diet, in addition to long-term chronic health effects, has its daily consequences, such as fatigue, apathy, lack of energy, disorders of processes related to brain function. Endocrine diseases are also common in such cases, which develop in secret for a long time, and one of the first visible symptoms may be menstrual disorders in girls (67).

The current study makes a valuable contribution to the literature on adolescents’ eating habits, particularly in the context of differences based on sex and BMI. It provides a deeper understanding of the relationship between these factors, an aspect that is often overlooked in similar research. In addition, the wide range of analyzed food categories, including both healthy and less favorable dietary choices, allows for a more comprehensive assessment of eating habits within the study group. An important feature of this study is its focus on snacking habits, which can significantly impact the overall energy and nutritional value of the diet. The analysis offers new insights into several key areas, including differences in snacking habits among children with different BMIs, a topic rarely addressed in the literature. Moreover, it identifies specific product groups that are more or less frequently consumed by children who are overweight, obese or malnourished. Another innovative aspect is the clear emphasis on the role of sex differences in shaping food choices among adolescents.

The findings of this study may contribute to the development of strategies for nutrition education and obesity prevention. They provide valuable data for public health organizations to use in health promotion activities targeting children and adolescents worldwide. Additionally, the study helps identify global nutritional trends among young people, serving as a foundation for developing international nutritional recommendations. The results can also support initiatives that promote healthy eating habits in schools, highlighting the importance of nutrition education and the impact of the family environment on children’s diet.

The study has several key strengths. The large and diverse sample size (664 children) enhances the reliability and representativeness of the findings. The inclusion of multiple variables, such as eating habits and socioeconomic factors, allows for a broader and more comprehensive interpretation of the data. An important aspect of the study was also the comparison of different food product consumption patterns based on sex and BMI, which helped identify key differences among the analyzed groups and draw more precise conclusions about the relationship between diet and nutritional status. In addition, the use of statistical methods, including the Kruskal-Wallis analysis of variance and chi^2^ test, strengthens the reliability of the results and ensures their objective interpretation. Another important aspect of the study is its practical application, as the findings can serve as a foundation for educational and preventive initiatives aimed at improving the dietary habits of children and adolescents.

This study also has several limitations. A notable limitation of the study is the absence of formal validation of the survey instrument and reliability testing. However, the researcher was present during the completion of the questionnaires and was available to respond to any inquiries. It did not account for the level of physical activity among adolescents, which is an important factor in assessing health and lifestyle. In addition, data on eating habits were collected through self-reporting, which may introduce recall bias or intentional modification of responses by participants. Certain socioeconomic aspects, such as family income or housing conditions, which could influence dietary habits, were not considered. Moreover, the study was conducted in a single region, which may limit the generalizability of the results to the broader population of children. It is crucial to acknowledge that the material used for publication was collected during the period 2016–2017. This must be taken into account when interpreting the results of the study. Another limitation is the age of the children studied. Despite these limitations, the study provides valuable insights that can contribute to international health programmers and initiatives aimed at improving the dietary quality of children and adolescents on a global scale. Moreover, the findings can be used by doctors to identify at-risk groups and implement appropriate interventions for overweight, obesity or malnutrition. Nutritionists and dieticians can use the study to develop more effective dietary strategies tailored to the needs of children and adolescents. Finally, the study is important for policymakers and public health institutions, providing a basis for prevention programmers and educational campaigns aimed at promoting healthy eating habits among young people.

## 5 Conclusion

Significant differences in the frequency of fast food consumption and snacking were observed between boys and girls, depending on BMI. The more frequent consumption of whole meal bread by overweight and obese adolescents, along with their lower intake of foods high in simple sugars and fast food, may suggest conscious attempts to control their diet, reflecting changing eating habits among young people. In contrast, the higher fast food consumption among malnourished girls, compared to other groups, may suggest a potential link between low body weight and a tendency to consume foods with low nutritional value.

The results of this study highlight the need for further research in this area. The application concerns the Polish teenage population. They also underscore the importance of nutrition education, particularly regarding proper meal composition and healthy snacking, tailored to specific groups based on BMI and sex. Special attention should be given to snacking habits, as they play a significant role in shaping young people’s diet. This study emphasizes the importance of an interdisciplinary approach that integrates nutrition, psychology and sociology. Promoting healthy eating habits should consider not only taste preferences but also the influence of family and the broader social environment. Thanks to integrated actions and the involvement of all sectors, it will be possible to reduce the scale of obesity in Poland and improve public health.

## Data Availability

The raw data supporting the conclusions of this article will be made available by the authors, without undue reservation.
